# Cryptococcal Meningitis and Anti-virulence Therapeutic Strategies

**DOI:** 10.3389/fmicb.2019.00353

**Published:** 2019-02-26

**Authors:** Kiem Vu, Javier A. Garcia, Angie Gelli

**Affiliations:** Department of Pharmacology, School of Medicine, University of California, Davis, Davis, CA, United States

**Keywords:** blood-brain barrier, *Cryptococcus neoformans*, meningitis, transcellular, paracellular, virulence

## Abstract

Fungal infections of the central nervous system are responsible for significant morbidity and mortality. *Cryptococcus neoformans* (*Cn*) is the primary cause of fungal meningitis. Infection begins in the lung after inhalation of fungal spores but often spreads to other organs, particularly the brain in immunosuppressed individuals. *Cn*’s ability to survive phagocytosis and endure the onslaught of oxidative attack imposed by the innate immune response facilitates dissemination to the central nervous system (CNS). Despite the success of *Cn* at bypassing innate immunity, entry into the heavily protected brain requires that *Cn* overwhelm the highly restricted blood-brain barrier (BBB). This is a formidable task but mounting evidence suggests that *Cn* expresses surface-bound and secreted virulence factors including urease, metalloprotease, and hyaluronic acid that can undermine the BBB. In addition, *Cn* can exploit multiple routes of entry to gain access to the CNS. In this review, we discuss the cellular and molecular interface of *Cn* and the BBB, and we propose that the virulence factors mediating BBB crossing could be targeted for the development of anti-virulence drugs aimed at preventing fungal colonization of the CNS.

## Introduction

The ability to grow at the host temperature separates *Cn* from other species of fungi that are unable to infect mammals. This acquired thermotolerance is a major virulence factor and the primary reason for *Cn*’s success as a pathogen. For reasons that are still not completely understood, *Cn* has a predilection for the brain making it the leading cause of fungal meningitis worldwide. Without early intervention, *Cn* produces self-contained cystic lesions (cryptococcomas) in the brain parenchyma, where fungal cells can replicate and thrive. These brain lesions compromise crucial areas of the brain tissue (Park et al., 2009; Williamson et al., 2017). Cryptococcal meningoencephalitis (CM), the leading cause of death among AIDS patients, has been observed in 20–60% of all solid organ transplant patients in the United States (Williamson et al., 2017). In 2014, HIV-associated cases of CM world-wide, of which ∼72.8% occurred in sub-Saharan Africa, was 223,100, culminating in 181,000 deaths (Rajasingham et al., 2017). Treatment guidelines for HIV-associated CM recommend amphotericin B with flucytosine for greater than 2 weeks as induction therapy; however, in Africa and Asia, where disease burden is the highest, access to flucytosine is nearly impossible (Loyse et al., 2013; Molloy et al., 2018). Even though patients in resource-poor settings have access to antiretroviral therapy (ART), the incidence of CM remains high (Tenforde et al., 2017).

Cryptococcal meningoencephalitis carries unacceptably high rates of mortality and even when treated, neurologic sequelae drastically reduces quality of life. This reality underscores the need for novel antifungal agents, especially ones with new mechanisms of action. However, fungi like *Cn* are eukaryotes, and that means very few pathogen-specific targets are available for antifungal drug development. This partly explains the exceedingly small arsenal of antifungal drugs, which is in stark contrast to the number of antibacterial agents. The old dogma has held the notion that antifungal agents must target viability, but several factors, including the limited number of antifungal agents, their limited efficacy and the emergence of resistance, are making it increasingly difficult to resolve fungal disease. The new way forward in the development of antifungals should encompass new targets that are associated with mechanisms of pathogenesis – i.e., anti-virulence agents (Dickey et al., 2017). These drugs differ from conventional therapeutics in that they do not affect the growth or viability of the organism and their efficacies are often more restricted. We propose that mechanisms of CNS penetration are a high-value target, and should be considered in the development of anti-virulence therapies.

## Mechanisms of CNS Invasion – Transcellular Migration

*Cryptococcus neoformans* harbors anti-phagocytic capabilities (Levitz et al., 1999) and a phagocytic-escape mechanism (Tucker and Casadevall, 2002; Alvarez and Casadevall, 2006) that facilitate undetected dissemination to the CNS. Upon moving freely within the bloodstream, *Cn* can lodge within the lumen of brain microcapillaries and cross the blood-brain barrier (BBB) directly via a transcellular mechanism (Chretien et al., 2002; Chang et al., 2004; Olszewski et al., 2004; Shi et al., 2010). The brain capillaries, and not the choroid plexus, appear to be the major route of entry as fungal cells were observed within the brain adjacent to capillaries 3 h post inoculation (Chretien et al., 2002; Chang et al., 2004; Shi et al., 2010). Real-time intravital imaging of mice following tail vein inoculation with *Cn*, revealed that freely moving fungal cells were rapidly sequestered by microcapillaries (Shi et al., 2010). The arrest of *Cn* in the microcapillaries of the brain appeared to be independent of viability or capsule size and similar to the movement of polystyrene microspheres, suggesting that *Cn* can become mechanically trapped within the microvasculature of the brain (Shi et al., 2010). The wedging of *Cn* within the smaller capillaries was initially reported in a previous study that performed extensive histological analysis of brain sections from mice inoculated via the trachea, to mimic the pulmonary route of infection, or inoculated intravenously (Olszewski et al., 2004). At 36 h post-inoculation, cryptococcomas were observed in diverse areas of the brain including the brain stem, colliculus, dentate gyrus, amygdala, cerebellum and lateral cortex/Ammon horns but not within the leptomeninx. Several studies have now confirmed that *Cn*, can cross brain microcapillaries and enter the perivascular space (Chretien et al., 2002; Chang et al., 2004; Olszewski et al., 2004; Charlier et al., 2009). The translocation of *Cn* from blood-to-brain begins with microcapillary sequestration that is promoted by the expression of urease (Olszewski et al., 2004). Once *Cn* crosses into the brain parenchyma, the growth and proliferation of *Cn* continued, resulting in cryptococcomas. This entire process can occur without the involvement of macrophages (Kozel and Gotschlich, 1982), suggesting that *Cn* can associate directly with the surface of brain microvascular endothelial cells (BMECs). Urease (Olszewski et al., 2004), Mpr1 (Vu et al., 2014), laccase (Qiu et al., 2012), phospholipase B1 (Maruvada et al., 2012), and a serine protease (Xu et al., 2014) have all been implicated in the dissemination of *Cn* to the CNS consistent with multiple fungal virulence factors mounting a coordinated effort.

Transcellular entry exploits the endocytosis pathway of the BBB. Recently, Aaron et al. (2017) found that EphA2, a tyrosine kinase receptor (TKR), was responsible for internalizing *Cn* and promoting transcellular crossing *in vitro*. An intriguing aspect of EphA2, is the notion that it may function as a general gateway for invasion of pathogens into host cells. Consistent with this notion, some species of viruses (Epstein-Barr virus) (Chen et al., 2018), bacteria (*Chlamydia trachomatis*) (Subbarayal et al., 2015), and the malaria parasite (Kaushansky et al., 2015) promote their entry into host cells by binding directly to EphA2. EphA2 belongs to the Ephrin (EPH) family of TKRs and along with their ligands, they make up the largest TKR subfamily (Kullander and Klein, 2002). EphA2 can affect cytoskeleton re-modeling and cell adhesion; in addition, studies have shown that of all the ten EphA-members, EphA2 is highly expressed in the brain endothelium and it can alter the permeability of the BBB (Carter et al., 2002; Kullander and Klein, 2002; Pasquale, 2005). The activation of EphA2 induces its dimerization and phosphorylation, triggering signaling events mediated by PI3K, MAPK, Src kinases, Rac1, and Rho-GTPases (Kullander and Klein, 2002).

RNA-seq analysis of human BMECs challenged with *Cn*, demonstrated that the EphA2-signaling pathway was significantly upregulated (Aaron et al., 2017). The study further demonstrated that transcellular migration of *Cn* across the BBB was dependent on EphA2 activity since knocking down EphA2 or blocking its activity prevented *Cn* from crossing the BBB. Also, *Cn* could phosphorylate- and colocalize with EphA2 in BMECs. The role of EphA2 was further supported by transient expression studies, where internalization of *Cn* was enhanced when EphA2 was overexpressed in HEK293T cells (Aaron et al., 2017). *Cn* did not appear to bind directly to EphA2 but instead, associated with CD44. This raises a compelling possibility – that *Cn* induces a cross-talk between CD44 and EphA2-signaling resulting in a permeable BBB via membrane and cytoskeleton remodeling (Aaron et al., 2017).

CD44, a surface glycoprotein located in lipid rafts/caveolae of endothelial cells, is the receptor for hyaluronic acid (HA), a component of the polysaccharide capsule that encases *Cn* (Zaragoza et al., 2009). *Cn*’s capsule is comprised primarily of glucuronoxylomannan (GXM) and galactoxylomannan (GalXM) and is a well-known virulence factor. Deletion of the capsule polysaccharide synthase 1 (*CPS1*) gene, that is responsible for the synthesis of HA, reduced the ability of *Cn* to associate with BMECs (Chang et al., 2006). HA concentration was found to be directly proportional to the ability of *Cn* to bind BMECs (Jong et al., 2007). HA interacts with the CD44 receptor on BMECs by initiating movement of actin to promote cellular entry into the endothelium (Jong et al., 2008b, 2012). Additionally, treating BMECs with simvastatin reduced CD44 levels and subsequently reduced fungal loads in the brain (Jong et al., 2012). Knockdown of CD44 in BMECs significantly reduced the adherence of *Cn*, and mice deficient in CD44 showed improved survival and less brain fungal burden (Jong et al., 2007, 2008a,b, 2012).

Several studies have noted that the cellular surface of BMECs becomes ruffled and forms F-actin projections upon exposure to *Cn* (Chen et al., 2003; Chang et al., 2004; Vu et al., 2013, 2014; Aaron et al., 2017). SEM and TEM studies revealed the formation of membrane protrusions on the surface of BMECs that fully engage *Cn* by wrapping around fungal cells and ultimately engulfing *Cn* (Chang et al., 2004; Vu et al., 2009). Rearrangement of actin filaments appears to play a crucial role during internalization since this likely produces the force required to generate the membrane structures that internalize *Cn*, similar to mechanisms involving other pathogens (Eugene et al., 2002; Nassif et al., 2002). The remodeling of actin filaments is mediated by small GTPases – RhoA, Rac1, and Cdc42, many of which have been shown to mediate transcellular crossing of *Cn* (Eugene et al., 2002; Kim et al., 2012). By inducing membrane-related changes, *Cn* facilitates its migration through a more permeable brain endothelium likely via the upregulation of endocytic-vesicles during transcellular migration.

## Paracellular and Trojan Horse Migration

As a facultative intracellular pathogen, *Cn* is able to survive and proliferate within the acidic environment of phagosomes. Given this ability, it is reasonable to suggest that *Cn* could co-opt phagocytic cells as a means to cross the BBB, via a Trojan Horse mechanism. Indeed some studies have demonstrated that this stealth-like mode of crossing lies within the tool box of *Cn*’s mechanisms of pathogenesis (Charlier et al., 2009; Sorrell et al., 2016; Santiago-Tirado et al., 2017; Kaufman-Francis et al., 2018). Using a static *in vitro* model of the BBB, one study found that phagocytes containing *Cn* could readily cross the barrier (Santiago-Tirado et al., 2017). *In vivo* studies revealed that mice inoculated with bone-marrow derived monocytes infected with *Cn* showed statistically more brain fungal burden compared to mice inoculated with free *Cn* at 24 h post-inoculation (Charlier et al., 2009). Furthermore, delayed and sustained phagocyte depletion in mice via clodronate injections, consistently reduced fungal burden in the brain and other organs (Charlier et al., 2009).

Recently, one study proposed that the perivascular space of the postcapillary venules, is the most likely site for phagocyte-dependent migration of *Cn* into the CNS (Kaufman-Francis et al., 2018). Once inside the perivascular space, free *Cn* released from phagocytes would migrate into the brain parenchyma by crossing the glia limitans (GL). The GL represents a second barrier that consists of astrocytic endfoot processes. Reactive astrocytes can regulate migration of leukocytes and humoral immune cells across the GL by forming tight junctions of their own thereby controlling entry into the CNS from the perivascular space (Horng et al., 2017). The formation of cryptococcomas beyond the GL support the notion that *Cn* crosses both barriers and uses different mechanisms to do so (Kaufman-Francis et al., 2018). Breaching the tight junctions of the astrocytic barrier could require fungal secreted proteases or local proteases such at MMP-2 and MMP-9 in the perivascular space (Horng et al., 2017; Kaufman-Francis et al., 2018). As the studies illustrate, the molecular basis for *Cn*’s migration to the CNS is multi-faceted and involves multiple virulence factors.

## The Anti-Virulence Approach

One of the main advantages of targeting virulence and not growth or viability is that, the compounds should not impose much selective pressure and therefore minimize the potential to induce antifungal drug resistance; thus, this will preserve the long-term use of antifungals and go a long way toward effective stewardship (Dickey et al., 2017). In addition, anti-virulence (AV) agents will not kill beneficial commensal populations, thus these new agents will very likely bypass this harmful side effect of anti-infectives. Targets mediating mechanisms of fungal pathogenesis also provide a new pipeline for drug discovery (Cui et al., 2015; Romo et al., 2017; Vila et al., 2017). They extend the range of potential drug targets from essential processes to virulence processes. This AV strategy is all the more relevant since the development of a lateral flow assay that can detect a cryptococcal antigen in under 10 min in symptom-free HIV-positive populations (Lindsley et al., 2011). Prior to this novel assay, patients suffering from cryptococcal infection, often presented with symptoms of CM, suggesting that cryptococci had penetrated the brain parenchyma; however, the lateral flow assay detects cryptococcal antigen in HIV-positive patients that do not demonstrate any cryptococcal-related symptoms, suggesting that AV compounds that block mechanisms of CNS penetration can be given pre-emptively or prophylactically to prevent the development of CM in vulnerable populations. Prophylactic administration of AV drugs in this case also gives the host a chance to mount a more effective immune response to keep cryptococci from disseminating systemically.

Although AV drugs can be an effective treatment on their own in mild cases, their inability to eliminate the pathogens likely necessitate simultaneous treatment with conventional drugs. Even though this may often be the case, the inclusion of AV agents alongside conventional therapy can reduce the dose of single drug usage with subsequent lower drug toxicity and reduced likelihood of resistance (Cui et al., 2015). For this reason, it will be necessary to examine how the AV compounds impact clinically relevant drugs such as amphotericin B, fluconazole, and flucytosine. It is also important to note that *in vitro* susceptibility testing of drug efficacy (i.e., determination of minimal inhibitory concentration) is not useful to determine the effectiveness of AV agents. These compounds will need to be examined for their ability to reduce the virulence of Cryptococcus in a more complex *in vivo* system.

## Anti-Virulence Drugs in the Clinical Setting

As a result of the ongoing antibiotic crisis and the slow pace of drug development, AV strategies have recently gained traction as a viable alternative approach despite their narrow spectrum of activity (Dickey et al., 2017). While the long term success of AV strategies remains to be seen, the use of AV therapeutics to treat bacterial infections have been demonstrated to be effective in the treatment of anthrax, botulism, and *C. difficile* infections (CDIs) (Arnon et al., 2006; Dawson et al., 2010; Tsai and Morris, 2015; Greig, 2016). For example, Bezlotoxumab (Zinplava) was approved to reduce the recurrence of CDI in patients who are at a high risk of recurrence (Dawson et al., 2010). The FDA has also recently approved two high-affinity mAbs, raxibacumab (Abthrax; GlaxoSmithKline) and obiltoxaximab (Anthim, ETI-204; Elusys Therapeutics) for the treatment and prevention of inhalation anthrax (Tsai and Morris, 2015; Greig, 2016). A comprehensive list of AV agents that are either in clinical trials or under advanced stage development has recently been reviewed by Dickey et al. (2017). In the coming years, many of these AV agents may prove invaluable to the treatment of bacterial and fungal diseases, especially in light of the present shortage of antibiotics, the increased rate of multidrug resistance, and the need to safeguard antibiotics for future use.

## Anti-Virulence Targets in Fungi

Due to the sheer volume of cases, it is not surprising that the vast majority of AV efforts to date have been directed toward treating bacterial infections (Dickey et al., 2017). While very little research has been done to develop AV agents for the treatment of fungal diseases, it is clear from the large body of literature in the bacterial fields that pathogenic mechanisms can be exploited to disarm pathogens and that AV agents have the potential to be invaluable additions to the current treatment regimes (Bicanic and Harrison, 2004). Although much of AV research in the fungal field is still in the early stage of drug discovery, recent work done in *Candida albicans* have identified several small molecules that inhibit pathogenic processes essential to *Candida*’s virulence (Cui et al., 2015; Romo et al., 2017; Vila et al., 2017; Mohammad et al., 2018). From their drug screen of 30,000 small molecule library, Romo et al. (2017) identified a novel series of bioactive compounds that prevented filamentation and inhibited biofilm formation – two major virulence attributes that are important for *C. albicans* pathogenesis, but dispensable for its growth and viability. The lead compound (N-[3-(allyloxy)-phenyl]-4-methoxybenzamide) provided protection against *C. albicans* infection in mouse models, strongly suggesting that this small molecule or its derivatives are promising candidates as AV agent to treat candidiasis. Aside from targeting *Candida* morphologic transitions for the development of AV agents, a recent 2018 study identified a novel Dibromoquinoline compound (4b) that targets metal ion homeostasis and exhibits anti-virulence activity at subinhibitory concentrations (Mohammad et al., 2018).

Among fungal pathogens, *Cn* is the most neurotropic and CM is the leading cause of CNS infection (Murthy and Sundaram, 2014). Several secreted virulence factors including phospholipase, urease and the metalloprotease, Mpr1, contribute to CNS invasion (Shi et al., 2010; Maruvada et al., 2012; Vu et al., 2014). Urease and Mpr1 are both present in fungi but absent in humans, making them promising targets for AV therapies (Rutherford, 2014). Mice treated with flurofamide, a urease inhibitor that has been proposed for clinical use, increased survival with significant reduction in brain fungal burden (Millner et al., 1982; Shi et al., 2010). Recently, several classes of urease inhibitors have been developed for the treatment of gastritis and gastric ulcer caused by *Helicobacter pylori* as potential AV agents (Macegoniuk et al., 2016; Liu et al., 2018). These compounds could be examined for their activities against *Cn* urease and further optimized for AV therapies. While urease is a well-studied virulence factor, Mpr1 has only recently been identified as an important extracellular protein promoting *Cryptococcus* invasion of the CNS (Vu et al., 2014). Mpr1 belongs to a distinct M36 class of fungalysins that are expressed in only some fungal species (Lilly et al., 2008; Vu et al., 2014; Na Pombejra et al., 2017). We demonstrated the importance of Mpr1 expression in BBB crossing of *Cn in vitro* and CNS invasion in a clinically relevant mouse model (Vu et al., 2014). We posit that Mpr1 represents another very attractive AV target to prevent cryptococci from breaching the CNS.

Aside from the contribution of secreted factors to CNS pathogenesis, surface-bound HA has also been shown to promote CNS invasion via binding to host CD44 receptors (Jong et al., 2007, 2008b). Treatment of cryptococci with 4-methylumbelliferone (Hymecromone), an approved drug known to inhibit HA synthesis, reduced binding of cryptococci to BMECs *in vitro* (Jong et al., 2007; Nagy et al., 2015). Because Hymecromone is an approved drug in Europe and Asia with a low toxicity profile, it has the potential to be used as AV agent to treat CNS infections caused by *Cn*.

Targeting virulence factors (VFs) that directly interact with or affect the integrity of the BBB for the development of AV agents is the most direct strategy to prevent *Cn* from breaching the CNS. However, it is also possible to target VFs that are important for *Cn* virulence during the early stage of infection. An in depth discussion of this strategy is, however, beyond the scope of this mini review. Three key VFs are important for *Cn* to establish infection in the lung: Phospholipase B1 (Plb1), laccase, and the polysaccharide capsule (Zhu et al., 2001; Santangelo et al., 2004; O’Meara and Alspaugh, 2012). Targeting pathways that regulate the expression of these VFs would make *Cn* vulnerable to intracellular killing by immune cells and prevent their systemic spread to the CNS. This strategy is, however, unlikely to be a viable solution. While Plb1, laccase, and the polysaccharide capsule are not essential for *Cn* viability, in the context of a mounted immune response, they are important for *Cn* to survive within the host. We expect that AV agents that inhibit the expression of Plb1, laccase or the polysaccharide capsule would likely promote *Cn* resistance. Indeed, resistance to AV compounds that target non-essential quorum sensing has been observed in *Pseudomonas aeruginosa* (Maeda et al., 2011). This cautionary tale underlines the need to take cell viability within the host into account for the selection of AV targets for drug development.

## Conclusion

Cryptococcal meningitis remains an important cause of morbidity and mortality in the immunosuppressed and HIV-infected populations. Despite decades of research, there remains a very limited repertoire of clinically relevant drugs. The lack of viable targets in fungal pathogens for drug development coupled with on-going threats of drug resistance makes the present dire public health situation more precarious. Targeting virulence factors rather than cell viability has shown promising results in the bacteria field and has opened up a new pipeline for drug development by greatly expanding the number of available targets. This new avenue of research is worth exploring and may represent one of several paths forward for the treatment of fungal infections, especially for cryptococcal meningitis.

## Author Contributions

AG conceived of the review and wrote and edited the review. KV and JG contributed to writing and editing the review.

## Conflict of Interest Statement

The authors declare that the research was conducted in the absence of any commercial or financial relationships that could be construed as a potential conflict of interest.
